# Commissureless Regulation of Axon Outgrowth across the Midline Is Independent of Rab Function

**DOI:** 10.1371/journal.pone.0064427

**Published:** 2013-05-16

**Authors:** Daan M. van den Brink, Oishik Banerji, Guy Tear

**Affiliations:** Medical Research Council Centre for Developmental Neurobiology, King’s College London, London, United Kingdom; Institut de la Vision, France

## Abstract

Nervous system function requires that neurons within neural circuits are connected together precisely. These connections form during the process of axon guidance whereby each neuron extends an axon that migrates, often large distances, through a complex environment to reach its synaptic target. This task can be simplified by utilising intermediate targets to divide the route into smaller sections. This requires that axons adapt their behaviour as they migrate towards and away from intermediate targets. In the central nervous system the midline acts as an intermediate target for commissural axons. In *Drosophila* commissural axons switch from attraction towards to extension away from the midline by regulating the levels of the Roundabout receptor on their cell surface. This is achieved by Commissureless which directs Roundabout to an intracellular compartment in the soma prior to reaching the midline. Once across the midline Roundabout is allowed to reach the surface and acts as a receptor for the repellent ligand Slit that is secreted by cells at the midline. Here we investigated candidate intracellular mechanisms that may facilitate the intracellular targeting of Commissureless and Roundabout within the soma of commissural neurons. Using modified forms of Commissureless or Rabs we show that neither ubiquitination nor Rab activity are necessary for the intracellular targeting of Commissureless. In addition we reveal that axon outgrowth of many populations of neurons within the Drosophila central nervous system is also independent of Rab activity.

## Introduction

During the development of the central nervous system a large number of neurons must form a precise pattern of connections. Each neuron extends an axonal process from its cell body that is able to interpret cues within its environment to navigate an often stereotypical route to identify and connect with its final target. Frequently neurons have to extend over a considerable distance and this process is facilitated by the neuron taking a series of smaller trajectories between intermediate targets or guideposts along their route. On reaching an intermediate target the axon must adapt its behaviour in order to leave and continue extending along its path. This adaptation may require changes to the receptors or their signalling ability at the leading edge of the neuron.

The cells at the midline of the developing central nervous system are a well-studied example of an intermediate target [1]. In all bilaterally symmetric organisms axons make a choice of whether to cross the midline or not. The majority of CNS axons are commissural axons which project from one side of the CNS to join a longitudinal tract on the opposite (contralateral) side, while a smaller number of axons never cross and remain on their own (ipsilateral) side. Those axons that cross the midline do so only once and do not re-cross. This suggests that shortly after the axon has reached the midline it adapts its response to the guidance cues it experiences such that it is no longer attracted to the midline.

The choice of whether to cross the midline is determined by the axon’s sensitivity to Slit, a repellent cue secreted by the midline cells. Axons that do not cross are sensitive to the Slit signal whereas the crossing axons are insensitive to the Slit signal. This sensitivity is dictated by the Roundabout (Robo) receptor that senses the Slit ligand as a repellent and directs axons away from the source of Slit [2], [3]. In mice and *Drosophila* Robo activity is high in ipsilateral projecting axons and in commissural axons after crossing the midline but low in commissural axons before they cross the midline [4], [5]. The *robo* mRNA is expressed continuously by all neurons from birth yet little or no Robo protein is present on the commissural axons as they head toward and across the midline [5] whereas those axons that remain ipsilateral express Robo on their cell surface continuously. Although the mechanisms involved in regulating Slit sensitivity differs in vertebrates and *Drosophila* both require the differential targeting of Robo proteins [6]–[8]. In *Drosophila* this process is directed by a short transmembrane protein, Commissureless (Comm) [6], [7], [9].

Comm is expressed by the commissural axons where it forms a complex with Robo and prevents Robo reaching the cell surface by directing Robo to an alternative vesicular location within the neuronal cell body [7], [9], [10]. Thus the decision whether to cross the midline is regulated by the presence or absence of Comm which controls whether an axon is sensitive to the Slit ligand via regulation of the intracellular location of Robo. Comm appears able to sort Robo from a pathway that delivers it to the cell surface into a pathway that maintains Robo within vesicles in the soma. Once the commissural axons cross the midline, Robo escapes Comm regulation and is able to accumulate on the surface of the distal regions of the axons. Despite our knowledge of the key role of Comm it remains unclear precisely how the targeting of Robo is achieved by Comm and how Robo escapes from Comm to reach the membrane within commissural neurons after crossing [11]. It has been demonstrated recently that in chick, like in *Drosophila,* Robo is held in an intracellular location within commissural neurons prior to midline crossing. Release of Robo from this location appears to be mediated by RabGDI which aids activation of Rab proteins to facilitate the trafficking of Robo to the cell surface [8]. Thus although vertebrates appear to lack a Comm orthologue, similar trafficking mechanisms may mediate the sorting of Robo in vertebrates and *Drosophila*.

Although it is well known that Comm is the component that sorts Robo to an intracellular location in *Drosophila* it is unclear what intracellular trafficking mechanism is utilised by Comm to target its localisation. Comm is targeted to an intracellular location in a variety of cells including *Drosophila* S2 cells and mammalian COS-7 cells or *in vivo* in embryonic muscles or neurons of the *Drosophila* CNS or PNS [7], [9], [12], [13]. When Robo is co-expressed with Comm they form a complex which is redirected to the same intracellular location as Comm alone. Thus the regulatory mechanisms that are able to target Comm are likely to be highly conserved and widely distributed in many different cell types. The identity of the trafficking pathways that direct Comm within commissural neurons remains unknown and the subject of this investigation.

## Materials and Methods

### Drosophila stocks

The following *Drosophila* stocks were used in this study: *comm* ^Δe39^ [14], *eagle-Gal4* [15], *elav-Gal4^C155^* [16], *RN2-Gal4* ^E^ (gift of M. Landgraf, University of Cambridge, UK), *UAS*-*Comm-Cherry* (this work), *UAS-myr-venus; eagle GAL4, UASCD8GFP*, *UAS-Hrs* (gift from H. Bellen, Baylor College, Houston, TX); *UAS-rab5GFP*, *UAS*-*rab7GFP*, *UAS-2XfyveGFP* (gifts from M. Gonzalez-Gaitan, University of Geneva, Switzerland); *UAS-GFP-shrub*, *UAS- GFP*-*spinster* (gifts from S. Sweeney, York University, UK); *UAS- commK>R* and UAS-*comm2PY>AY –HA* (gifts from D. Rotin, University of Toronto, Canada). *UAS-YFPrab3^T35N^*, *UAS-YFPrab4^S22N^*, *UAS-YFPrab5^S43N^*, *UAS-YFPrab5^Q88L^*, *UAS-YFPrab7^T22N^*, *UAS-YFPrab7^Q67L^*, *UAS-YFPrab11^S25N^*, *UAS-YFPrab23^S51N^*, *UAS-YFPrab26^T204N^ UAS-YFPrabX4^T40N^* [17] *UAS-shi^K44A^* and *UAS GFP-Clathrin Light Chain*, were obtained from the Bloomington Stock Centre. A description of genetic markers and chromosomes can be found at FlyBase (http://flybase.bio.indiana.edu).

### Molecular Biology

Robo and Comm were amplified by PCR using an embryonic cDNA library as template and were cloned into pENTR/D-TOPO (Invitrogen) according to manufacturer’s instructions. To obtain the Comm-mCherry construct, mCherry was amplified by PCR from a mCherry-pRSET-B plasmid (gift from R. Tsien) [18] using a 5′ primer containing a Bsu36I site, while Comm coding sequence was amplified with the same site at the 3′ end. The constructs were subsequently combined by restriction using EcoRV (present in the pENTR vector) and Bsu36I yielding an in-frame Comm-mCherry-pENTR vector. The pENTR constructs were cloned into Drosophila expression vectors from the Drosophila Gateway Vector Collection by LR Recombination with Gateway LR-clonase enzyme (Invitrogen). The vectors contain an Actin5C or Gal4-responsive UAS promoter with either an N-terminal Myc- or Venus-tag, depending on the intended use.

UAS-Comm-mCherry transgenic flies were obtained by standard P-element mediated germ line transformation by BestGene Inc.

### Cell culture, transfection and immunofluorescence

Schneider S2 cells (Drosophila Genomics Resource Centre) were cultured and transfected in serum free Insect Express Sf9-S2 (PAA Laboratories Ltd). A Robo-Venus construct driven by an Actin promoter and a Comm-myc construct driven by a Metallothionein promoter (pRmHA-Comm-myc [9]) were co-transfected at 1μg each, using Cellfectin II reagent (Invitrogen) according to the supplied protocol. Briefly, 10^6^ cells were plated on a coverslip coated with Concanavalin A in 6 well plates and allowed to attach overnight. After transfection cells were left to recover after which expression of Comm was induced by adding 0.1 mM CuSO_4_ and incubated for 16 hours. Cells were then washed in PBS and fixed in 4% formaldehyde with 0.1% Triton in PBS for 20 minutes, blocked in 100 mM ammonium chloride in PBS for 10 minutes, washed 3 times in 10 mg/ml bovine serum albumin in PBS and then incubated with primary (9E10, Santa Cruz and anti-GFP, Invitrogen) and fluorescent-secondary antibodies (Invitrogen). Coverslips were mounted in Vectashield (Vector Laboratories) and imaged on a Zeiss Axioplan 2 fluorescent microscope.

### Immunostaining and live imaging

Embryos were collected overnight, dechorionated in 50% sodium hypochlorite and rinsed in distilled water. For immunostainings, the embryos were processed according to [19] and imaged on a Zeiss LSM 510. For live imaging, embryos were mounted in Voltalef halocarbon oil (Atofina) on a hydrophobic, gas-permeable membrane of a Lumox dish (Sarstedt) and confocal stacks were recorded on an UltraVIEW VoX (Perkin Elmer) spinning disk system coupled to a Zeiss Axio Imager M1 microscope.

Image processing and analysis was done using Volocity software (Perkin Elmer). Objects were identified in 3 dimensions for each channel by determining threshold intensity deviations of means. The recognised vesicles were subjected to a function for separating touching objects using analyses of shape and overlap of unique objects. Objects below a size threshold of 0.4 µm^3^ (corresponding to 5 voxels or less) were not considered valid vesicles but background noise. Overall, the objects identified by the software corresponded well with an unbiased human observation.

To quantify colocalisation the number of objects with overlapping signal in the two channels were counted. Minimum threshold intensities were adjusted between experiments to allow for variations in background fluorescence, only objects with significant signal in both channels were counted as colocalised.

## Results

### Comm activity in commissural axons does not require Nedd4 mediated ubiquitination

In commissural neurons Comm acts to sort the Robo receptor protein into intracellular vesicles within the soma and prevents its passage to the cell surface thus rendering these axons insensitive to the Robo ligand, Slit. It has been strongly suggested that Comm is sorted directly to its intracellular location in commissural neurons while in muscles Comm may be endocytosed from the plasma membrane [13], [20]. Previous studies by us and others have surveyed the activity of deleted forms of Comm and identified that a ’sorting signal’ between amino acids 220 and 244 is necessary for its localization and Robo sorting function. This region contains the motifs PPCY and LPSY which form a binding site for Nedd4, an E3 ubiquitin ligase. Nedd4 catalyses the ubiquitination of Comm [9], [21], [22] and monoubiquitination can act as a signal to internalise membrane proteins [23]. Mutation of the Nedd4 binding site (Comm2AY) in which the PPCY and LPSY motifs are mutated to AY or replacement of all the intracellular lysines in Comm with arginine (Comm10K->R) to remove all the sites available for ubiquitin addition, diverts localisation of Comm to the cell surface of S2 cells and muscles [9], [13]. However, it has been suggested that Comm activity in commissural axons does not require Nedd4 or ubiquitination [12]. To confirm the observations in the CNS we used an overexpression assay to examine the ability of these Comm variants to downregulate Robo activity.

**Figure 1:**
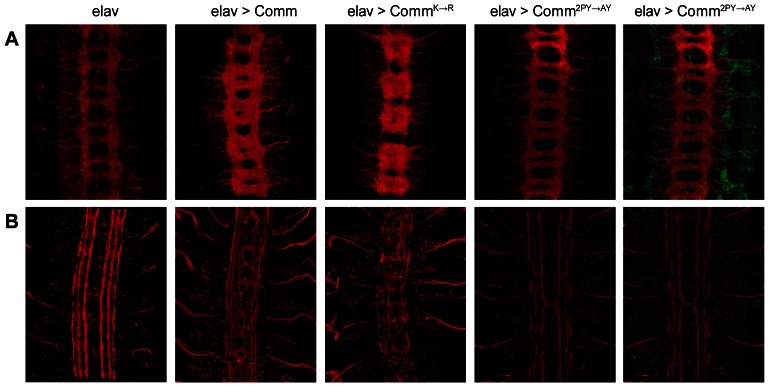
Comm activity is dependent on PPCY/LPSY motifs but independent of ubiquitin attachment. Immunostainings of the *Drosophila* embryonic central nervous system to reveal the activity of the indicated UAS-Comm constructs when expressed pan-neuronally by elav-Gal4. Comm is the wild type allele, CommK>R is a construct where all intracellular lysines have been mutated to arginines to prevent ubiquitination and Comm2PY>AY contains mutations in both PPCY and LPSY motifs. A) CNS axons in stage 15 embryos visualised with the BP102 antibody. B) Longitudinal axons in stage 16 embryos within the CNS revealed with MAb 1D4. The right-most panels in A and B show co-staining (green) of the HA-epitope present on the Comm2PY>AY construct to confirm its expression by elavGal4.

When Comm is over-expressed in all neurons using the elav-Gal4 driver, Robo activity is reduced and ipsilateral axons are re-routed across the midline and display a *robo* phenocopy as visualised using a monoclonal antibody, BP102, which recognises the majority of CNS axons (Figure 1). Overexpression of Comm2AY did not cause any change to the normal orthogonal pattern of axon outgrowth while driving Comm10K->R resulted in a *robo* phenocopy equivalent to that generated by overexpressing the normal Comm protein. As Comm10K->R retained full Comm activity to reroute CNS axons, this confirmed that ubiquitination of Comm is not necessary for Comm function to regulate Robo activity in CNS neurons. However, the inability of Comm2AY to generate a phenotype suggests that the PPCY/LPSY motifs are essential for Comm activity as we also suggested previously [9]. Since ubiquitination is unnecessary for Comm activity it is likely that this region comprises an essential binding site for other molecules in addition to Nedd4 that are necessary for the intracellular trafficking of Comm and its ability to relocalise Robo.

### Comm co-localizes with late endosomal markers

Comm sorting to an intracellular compartment is essential for Comm function and this is not mediated by Nedd4 within commissural neurons [12]. Previous studies investigating the distribution of Comm within COS7 cells have identified that Comm is localized within a late endosomal compartment. We investigated the localisation of Comm within commissural neurons by analysing the distribution of Comm with respect to that of individual Rab proteins and other compartment markers using time lapse microscopy.

**Figure 2:**
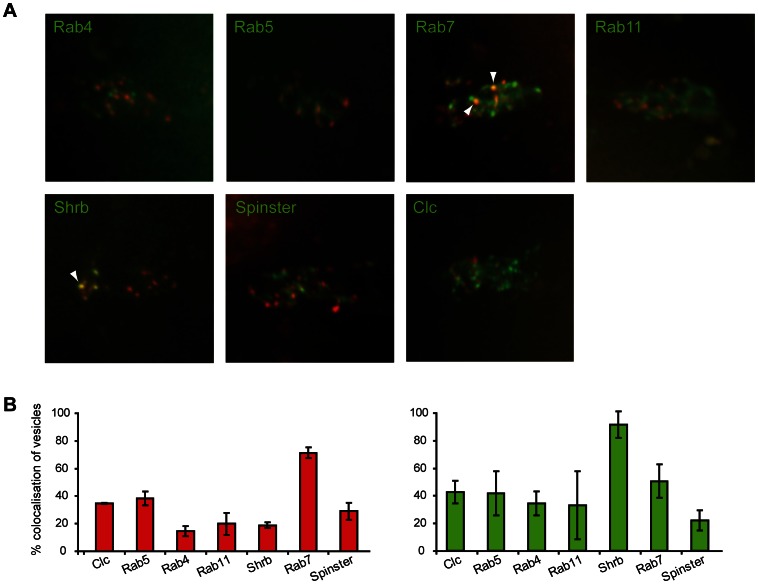
Localisation of Comm expression within commissural neurons with respect to intracellular compartment markers. A) Confocal images of Comm-mCherry and different intracellular compartment markers, labelled by GFP or YFP, expressed in eagle-neurons of stage 15 embryos. Constructs contain an UAS element and were driven by the egGal4 line, which drives expression in a specific subset of commissural neurons [15]. The cell bodies of a single EG cluster is shown in each panel. Comm colocalises with Rab7 and Shrub (arrowheads). B) Quantification of the vesicle pool that shows colocalisation between the red and green channels. (i) Red bars indicate the percentage of Comm vesicles that are positive for a specific marker out of the total pool of Comm vesicles. (ii) Green bars indicate the percentage of vesicles that express a specific marker which also overlap a Comm vesicle out of the total pool of marker vesicles. The percentages of colocalisation of firstly Comm with Rab7 (red bars) and secondly Shrub-GFP with Comm (green bars) are significantly different from each other compartments in their respective groups (at least P<.01, one-way ANOVA with a Tukey-Kramer post test).

We used the eagle-GAL4 (eg-GAL4) driver to drive expression of markers for intracellular compartments within a subset of commissural neurons together with Comm tagged with mCherry (Figure 2A). This driver drives expression in a small number of commissural neurons that extend axons within the anterior and posterior commissures in the Drosophila embryo [24]. The markers we used were GFP-tagged Rabs together with GFP-Shrub, GFP-Spinster and GFP-Clathrin Light Chain(Clc) and these were coexpressed individually with Comm. Rab4 and Rab11 associate with recycling vesicles, Rab7 is a marker for late endosomes, Clc and Rab5 are markers for early endocytic vesicles, Shrub (the orthlogue of mammalian CHMP4) is part of the ESCRT-III complex in the sorting endosome and Spinster is a lysosomal membrane protein. Expression of Comm and the tagged markers were monitored in real-time within the soma of the eagle-neurons during stage 15. We calculated the percentage of the Comm vesicles that co-localised with a compartment marker (Figure 2B) and the percentage of vesicles bearing a particular marker that also contained Comm (Figure 2C). Comm did not show complete co-localisation with any of the markers rather a partial co-localisation with several, suggesting that Comm may move through a number of compartments. We did observe instances of apparent movement of Comm into and out of a specific Rab compartment. When examining the Comm vesicles these were most extensively co-localised with Rab7. 70% of the Comm vesicles also contained Rab7, when Rab7-GFP was expressed with Comm. However the number of Comm vesicles only account for 50% of the population of Rab7 vesicles, suggesting that Comm is not solely targeted to this compartment.

Comm is consistently found within the vesicles marked by Shrub-GFP, over 90% of the vesicles that contain Shrub-GFP also express Comm. The Shrub vesicles only comprise a small number of vesicles in the commissural neurons which account for 20% of the Comm containing vesicles, suggesting Comm is not exclusively localised to these vesicles. This distribution of Comm indicates that Comm appears to transit through a variety of intracellular locations but exists predominantly in a sorting or late endosomal compartment.

### Blocking Rab function does not disrupt Comm function or axon outgrowth in the Drosophila CNS

We find that Comm is most commonly found associated with the Rab7 late endosomal compartment in commissural neurons. Comm may not be sorted directly to this compartment as it is also found associated with a number of other membrane compartments, or Comm may transit from the Rab7 vesicles to other vesicle populations as part of its normal activity. Since Comm appears to accumulate within the Rab7 compartment this suggested to us that Rab7 might play a role in targeting Comm within commissural neurons. To examine if Comm localisation to or through the Rab7 compartment is essential for its activity we disrupted Rab function in commissural neurons using dominant negative forms of the Rabs.

**Figure 3:**
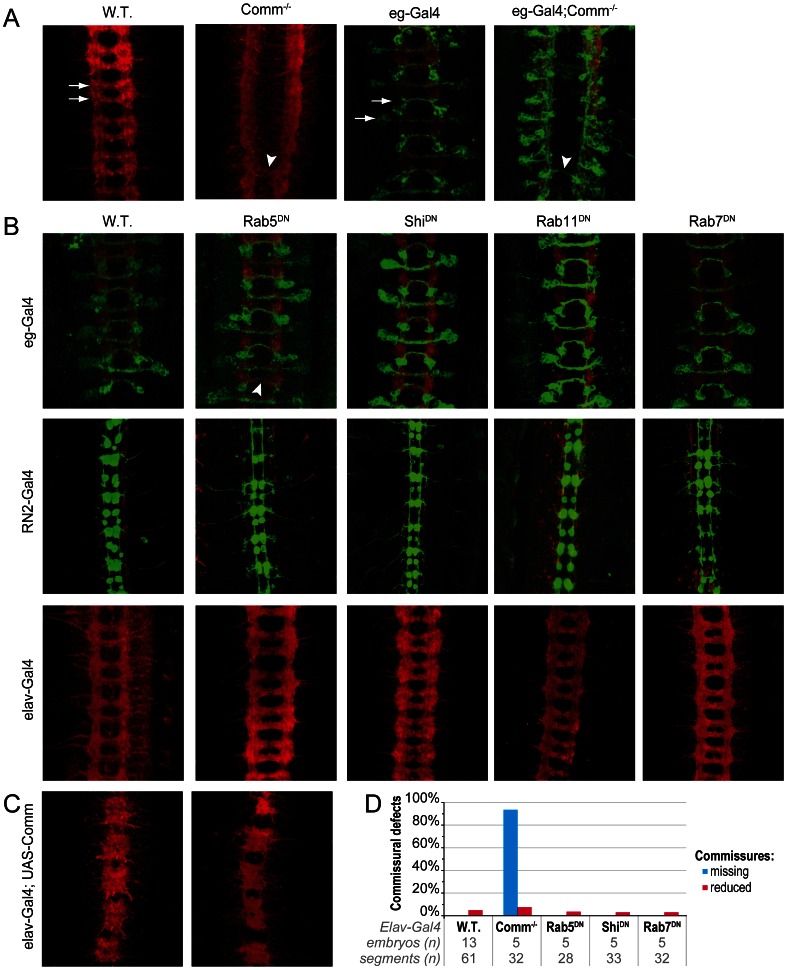
Axonal guidance is not affected by expression of dominant negative regulators of endosomal trafficking. A) Comm is required for the formation of commissures. BP102 staining (red) reveals the normal pattern of axon pathways in the wild type *Drosophila* CNS (red). Commissural neurons (arrows) fail to form in the majority of segments in a *comm* mutant although the occasional commissure may form (arrowhead). Eg-Gal4 driving expression of CD8::GFP reveals a subset of commissural neurons (green) which fail to cross the midline in *comm* mutant embryos. B) Drosophila embryos were obtained from crosses of indicated dominant negative constructs with driver lines for expression in commissural (eg-Gal4), longitudinal (RN2-Gal4) neurons or with a pan-neuronal driver (elav-Gal4). The lines RN2- and eg-Gal4 also express CD8::GFP to enable identification of axons. Embryos were stained with anti-GFP (green) and counterstained (red) with either BP102 or 1D4 (in the case of RN2-Gal4). RN2-Gal4 neurons drive expression in longitudinally projecting eve-neurons [25]. Expression of the dominant negative constructs using these drivers does not disrupt axon outgrowth and guidance although some minor defects do occur (arrowhead). C) Expression of Comm in elav-neurons results in a *roundabout* phenocopy where longitudinal neurons cross the midline. This gain of activity of Comm is not affected by the co-expression of Rab5^DN^ suggesting Rab5 is not required for Comm activity. D) Quantification of the ability to form commissures in *comm* mutant embryos or embryos expressing dominant negative forms of Rab5, Rab7 or Shibire reveal that commissural neuron outgrowth is independent of Rab5, Rab7 and Shibire activity.

In the absence of Comm commissural axons fail to extend across the midline and extend within the longitudinal pathways as revealed by both BP102 staining and by expression of membrane targeted GFP in the eagle neurons (Figure 3A). We anticipated that blocking the activity of the Rabs would disrupt the trafficking of Comm and its activity so disrupting the ability of axons to extend across the CNS midline similar to when Comm is absent. We found that driving expression of a dominant negative form of Rab7^DN^ (T22N) in the eagle positive commissural neurons did not disrupt their outgrowth across the midline (Figure 3B). Similarly expression of a constitutively active form of Rab7^ CA^ (Q67L) did not affect extension of commissural neurons and the CNS forms normally. To identify if the outgrowth of other axonal populations might be sensitive to a disruption of Rab7 activity we expressed Rab7^DN^ in longitudinal axons using the RN2-GAL4 [25] driver or in all neurons using the elav-GAL4 driver. In both cases expression of Rab7^DN^ did not result in any defects in axon outgrowth. To see if other vesicle trafficking pathways were required for Comm activity or for normal axonal outgrowth, we used the same three drivers to express other dominant negative Rabs. Neither expression of Rab4^DN^ or Rab11^DN^ caused any abnormalities in axon outgrowth when expressed with any of the drivers. We also co-expressed Rab4^DN^ and Rab11^DN^ to overcome possible redundancy between these Rabs but still no phenotype was observed (data not shown). In addition overexpression of Hrs, which leads to enhanced trafficking of receptors from the cell surface to MVBs and lysosomes [26], did not result in any phenotypes. Similarly axonal outgrowth was not affected by expression of Rab5^ CA^ or Rab11^CA^.

Finally we expressed dominant negative constructs of Shibire (the *Drosophila* dynamin homologue) and Rab5 in the developing central nervous system to disrupt membrane supply from the cell surface. Again when either construct, Rab5^DN^ or Shi^DN^, were expressed in eg-neurons, extensions of axons across the commissures were normal. Furthermore, neither construct had any influence on the extension of axons along the longitudinals when expressed in RN2 neurons or any effects on CNS development when using elavGal4 as a pan-neuronal driver. In addition we tested whether any of the Rabs that have recently been described to be expressed specifically in the *Drosophila* nervous system [17]are required for axon outgrowth in the embryonic nervous system. However, overexpression of dominant negative forms of Rab3, Rab23, Rab26 or RabX4 by any of the three drivers did not result in an axon outgrowth phenotype.

We also examined whether the midline crossing phenotype that results from an overexpression of Comm in the ventral nerve cord could be a more sensitive assay for Rab function. When Comm is overexpressed in neurons Robo fails to reach the cell surface and many axons extend across the midline. Potentially this phenotype may be more sensitive to changes in the proteins necessary for Comm activity as these would be required at an increased level and dominant negative Rab constructs may reduce the severity of the comm overexpression phenotype. However when RN2-Gal4 was used to drive expression of Rab5^DN^ together with Comm, no reduction in aberrant crossing of the midline was observed (data not shown). A similar result was found for elav-Gal4, where the level of Comm mediated redirection of ipsilateral projections occurred to the same extent in the presence or absence of Rab5^DN^ (Figure 3).

**Figure 4:**
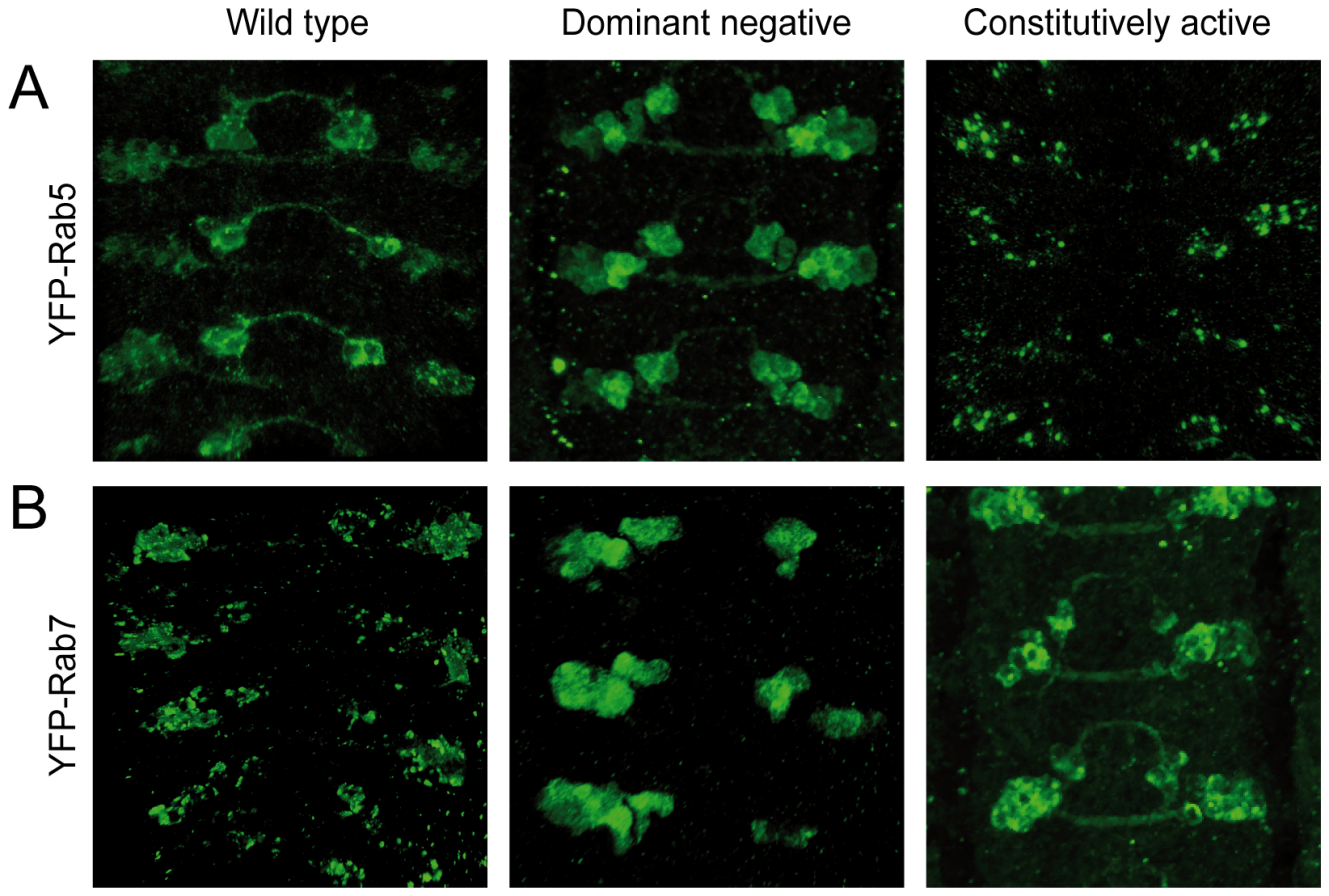
Vesicle size is perturbed in commissural neurons by dominant negative forms of Rab5 and Rab7. Expression of wild type, constitutively active or dominant negative forms of Rab5 or Rab7 lead to changes in the size and distribution of vesicle compartments within commissural neurons. When a dominant negative form of Rab5 is expressed the Rab 5 compartment becomes diffuse while a constitutively active form drives the formation of larger more punctate compartments. Similarly interference with Rab7 changes the appearance of the Rab7 vesicles.

To verify the Rab constructs were functional, we tested their efficacy in the eye and wing since Rab function is required in these tissues for their normal development [27], [28]. As expected, expression of the Rab constructs with GMR-Gal4 and en-Gal4 resulted in patterning defects confirming their activity (data not shown). To demonstrate that they were also active within the embryonic CNS we investigated whether the constructs lead to changes in the morphology of the vesicles they associate with as previously reported for dominant negative and constitutively active forms of the Rabs [17]. When expression of either Rab5^CA^ or Rab7^CA^ was driven by egGal4, a clear increase in vesicle size is seen, while expression of their dominant negative forms result in a more dispersed localisation (Figure 4). Thus interference with Rab function in the CNS axons did disrupt elements of the normal vesicle trafficking in the developing neurons as they navigate the CNS midline yet axonal outgrowth and guidance is unaffected.

### Effect of Rab mutants on Comm and Robo trafficking in S2 cells

**Figure 5:**
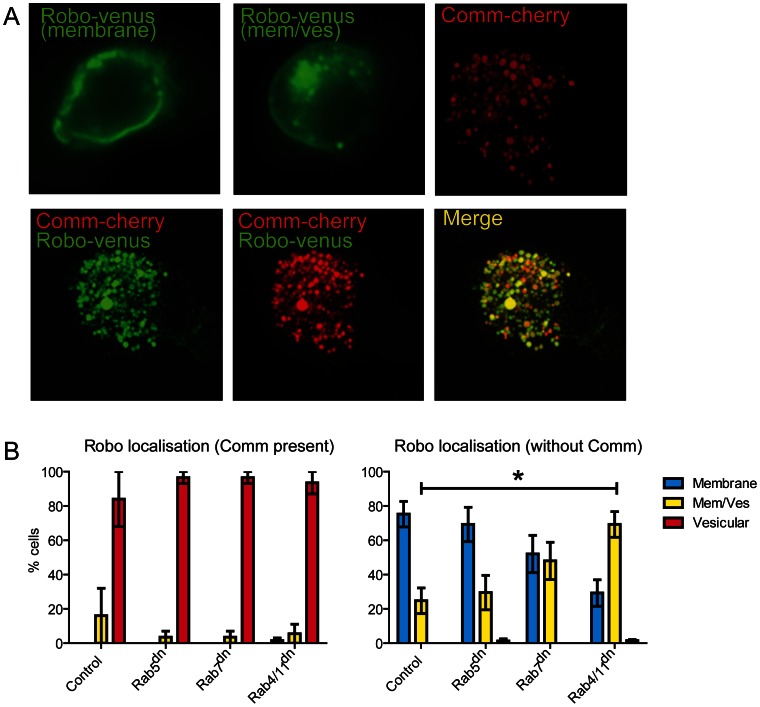
Effects of reduction of Rab activity on localisation of Comm and Robo expressed in S2 cells. A) Representative images of Robo-Venus and Comm-mCherry in transfected S2 cells (for details see Material and Methods). The top panels show transfected cells with tagged version of either Robo or Comm. Robo is predominantly localized on membranes, but in some cells an additional vesicular component can be observed (indicated ‘membrane’ and ‘mem/ves’). Comm is always present in vesicles. The bottom panels show a co-transfection of Robo with Comm resulting in a complete exclusion of Robo from the plasma membrane. B) Quantification of the effect of reducing Rab function on the localisation of Robo in S2 cells in the presence or absence of Comm. Dominant negative constructs of Rab5, Rab7 or Rab11 have no effect on the Comm driven relocalisation of Robo. However, when Comm is not present, a reduction of Rab11 and Rab4 leads to a failure to accurately target Robo to the cell surface and Robo is found within intracellular vesicles (p<0.05).

As interference with Rab function in *Drosophila* embryonic neurons did not have an effect on axon outgrowth, we investigated whether the Rabs are necessary in S2 cells for the normal localisation of Robo or the ability of Comm to relocalise Robo (Figure 5). Venus-tagged Robo is directed predominantly to the cell surface in S2 cells with 24% of cells also showing some Robo within vesicles. This distribution is not significantly changed when Rab5^DN^ is co-expressed with Robo-Venus. When Rab7^ DN^ is co-expressed, however, the distribution of Robo appears to shift slightly with a reduction of Robo on the cell surface and more within vesicles, suggesting interference with Rab7 results in a less efficient trafficking of Robo to the cell surface. When Rab4^DN^ and Rab11^DN^ are co-expressed in the S2 cells with Robo-Venus, there is a significant change in Robo distribution. Rab4^DN^ and Rab11^DN^ block recycling of internalised plasma membrane proteins leading to their build-up in the recycling compartment. This suggests that normally a portion of Robo is being recycled to and from the cell surface. Thus normal activity of the Rab proteins is required to efficiently traffic and maintain Robo at the cell surface in S2 cells.

When Comm is co-expressed with Robo-Venus in S2 cells the Robo protein is redirected from the cell surface to a vesicular location together with Comm. This localisation is dependent on the ‘sorting’ signal located between amino acids 220 and 244, since variants of Comm with this signal deleted or mutated are localised at the cell surface [9], [12]. Introduction of Rab5^DN^, Rab7^DN^, or a combination of Rab4^DN^ and Rab11^DN^ into the S2 cells with Comm and Robo-Venus does not affect the sorting of Comm and Robo to their intracellular localization. These results strongly suggest that the intracellular trafficking of Comm is independent of Rab function and that association with Comm can separate Robo from Rab mediated trafficking mechanisms within the cell.

## Discussion

Axon outgrowth is a highly coordinated process that requires neurons make pathfinding decisions as they navigate from their place of birth to their final cellular target. These pathway choices can result from axons responding to new cues they encounter in their environment as they advance or a change in the axonal response to environmental cues they have previously met. Such changes in axon sensitivity can result from modulation of downstream signalling or changes to the repertoire of receptors on the surface of the extending axon [29], [30]. The mechanisms which control distribution of guidance receptors within neurons is becoming subject to increased attention [31]–[33]. There is considerable evidence that cell surface molecules are directed to particular regions of the extending axon and that axonal behaviour can be influenced by adapting the trafficking of individual guidance proteins [31]. Little is known regarding the trafficking of molecules during early development of the nervous system and the intracellular components that might regulate this trafficking [32].

Switches in axon behaviour occur as the axons reach and leave intermediate targets en route to their final target. A well-studied switch in axonal behaviour occurs at the midline of the nerve cord [1], [34], [35]. Commissural axons are initially attracted to the midline by Netrin, and other attractive cues, however once they reach the midline axons lose their sensitivity to Netrin and become sensitive to Slit, a midline repellent cue. This change in sensitivity is a result of an increase in the levels of Robo receptor on the axonal cell surface which recognises the Slit ligand as a repellent and, in rodents at least, can reduce the activity of DCC, the Netrin receptor [36]. In both *Drosophila* and vertebrates Robo protein levels are low on commissural axons prior to crossing the midline but increase as axons reach and cross the midline [4], [5]. In *Drosophila* commissural neurons, levels of cell surface Robo protein are regulated by Comm which binds to Robo and prevents its accumulation on the cell surface by redirecting Robo to the same intracellular location occupied by Comm [7], [9]. Although the function of Comm has been known for some time it is unclear how Comm is targeted and so how it re-directs the trafficking of Robo. Since Comm can redirect Robo in a variety of cell types it has been assumed that Comm utilises a conserved mechanism to reach its intracellular destination.

### Comm is associated with late endosomes in commissural neurons

Here we have attempted to identify the trafficking components that may direct Comm and hence Robo within the commissural neurons. We first examined the nature of the intracellular location of Comm in commissural neurons by using time lapse microscopy to examine the location of Comm relative to a number of tagged proteins that identify specific compartments in the cell. This analysis revealed that Comm is found associated with multiple locations within the neuronal soma but is predominantly found to co-localise with Rab7 and thus associated with the late endosome. Comm was also found to show some overlap with each of the markers we used suggesting that it is trafficked through a variety of compartments prior to reaching the late endosome. In addition in the time lapse studies we were able to visualise Comm moving in and out of the Rab 7 vesicles suggesting that Comm moves between compartments. When we examined the percentage of vesicles of a particular compartment that contained Comm we observe that the majority of the Rab7 vesicles contain Comm while all the vesicles identified by Shrub-GFP contain Comm. Shrub is the fly homologue of the ESCRT-III complex protein CHMP4/Snf7 that is associated with maturation of late endosomes potentially to multivesicular bodies (MVB) [37]. Each eg-neuron contains a small number of Shrub-GFP vesicles that all contain Comm, while Comm is also found in additional vesicles in the same cell. Thus Comm appears to be directed into a late endosomal compartment where it may be retained or shuttled to a MVB compartment.

These late endosomes are mainly restricted to the soma and very few Comm vesicles migrate into the commissural axons. It is unclear whether Comm is maintained in the late endosomal compartment in commissural neurons or if it is transferred to the lysosome for degradation along with any binding partners or potentially to the MVB. When Comm is expressed in peripheral neurons such as those identified by the pox-N GAL4 driver, we observed as have others that Comm is able to migrate into the axons [12], suggesting a potential restriction on Comm trafficking within commissural neurons. This localisation of Comm, and thus Robo, to Rab7 endosomes, appears to be different to where Robo is targeted within chick commissural neurons. Chick Robo appears to be targeted to Rab11 positive vesicles, revealing that despite some similarities in Robo trafficking, the mechanism which prevents Robo from reaching the cell surface prior to midline crossing differs between vertebrates and *Drosophila.* In chick Robo is released from being held in its intracellular location by RabGDI, which stimulates activity of the RabGTPases [8]. We also find that the activity of Rab7, 4 and 11 is necessary for Robo to reach the cell surface in *Drosophila* cells. However when Robo is associated with Comm, interference with Rab function has no influence on the ability of Comm to retain Robo within intracellular vesicles. It would be of interest to identify if RabGDI has a similar role in *Drosophila* commissural neurons to allow the transfer of Robo to the cell surface when Comm is downregulated during midline crossing.

### Targeting of Comm within commissural neurons appears to be independent of both ubiquitin mediated targeting and Rab activity

Previous studies have demonstrated that a ‘sorting region’ between amino acids 220 and 244 is essential for the intracellular targeting of Comm and its ability to redistribute Robo within commissural neurons. Comm variants missing this region are localised to the cell surface. This region includes a binding site recognised by the WW domains within the ubiquitin ligase Nedd4 and binding of this region by Nedd4 can lead to the ubiquitination of Comm, leading to the suggestion that Comm localisation may be mediated by an ubiquitin dependent process. However prevention of Comm ubiquitination by removal of the lysines necessary for ubiquitin attachment does not abolish Comm’s ability to generate a *robo* phenocopy. This suggests that other regulatory proteins bind this region. Although the sequence does not contain any other recognised motifs, there exist additional WW domain-containing proteins that could be candidates to bind this region. The same PPxY motif is also found within Hrs/vps25, for instance, and is associated with the ESCRT-II binding complex known to be important for endosomal trafficking. Yet overexpression of Hrs failed to interfere with Comm activity.

As Comm is principally directed to a Rab7 positive late endosomal destination and appears to be directed there via an association with other compartments marked by other Rab proteins we tested whether interference with the Rabs would block the trafficking of Comm, thus preventing Comm from being able to regulate Robo levels in pre-crossing axons and so lead to an axon outgrowth phenotype. However our manipulations of Rab function in commissural neurons did not disrupt their ability to navigate across the midline despite a clear change to vesicular morphology within the neurons. More intriguing is our observation that expression of dominant negative or catalytically active forms of the Rabs did not impede the ability of any neurons to extend within the CNS. Our observations suggest that the Rab proteins might not be necessary for axon advance or guidance during the early development of the Drosophila CNS.

### A redundant role for the Rabs in endosomal trafficking in axon extension

Trafficking of cell surface molecules via endosomes has been suggested to be required during neuron extension to direct the transport of cell adhesion molecules along axons or during regulated endocytosis and exocytosis at the growth cone to control responsiveness to specific ligands [29], [31]–[33], [38]–[40]. For example levels of L1/NgCam, β1-Integrin and Sema3A in axons and at the growth cone are known to regulated by endocytosis and/or intracellular vesicle trafficking [41]–[43]. The precise intracellular mechanisms that target these proteins to endosomes and control their distribution are not fully understood. Although the Rabs are strong candidates to play a role in these processes, there have been few tests for their involvement to regulate axon outgrowth *in vivo*. Rab11 has been revealed to be necessary for the trafficking of β1-Integrin in dorsal root ganglion neurons *in vitro* [43]. Knockdown of Rab11 levels within primed PC12 cells does lead to decreased neurite outgrowth [43] and Rab8 depletion in cultured hippocampal neurons has been shown to also reduce extension [44], yet these requirements have not been demonstrated *in vivo*. One previous study has indicated a potential requirement for Rab11 for axon outgrowth in *Drosophila* [45] however the phenotypes are very mild and we did not observe any major defects using similar reagents. Essential roles for Rabs or their effectors have been revealed at the synapse or for dendrite extension [46]–[49] and it is thought that the there are differences between axonal and somatodendritic endosomes [50] and potentially the Rabs we investigated here may have a lesser role in axon outgrowth.

It remains unclear which mechanisms traffic Comm to its final destination within commissural neurons as it appears to be independent of many Rabs and doesn’t require ubiquitination. Potentially members of the ESCRT pathways that are required for intracellular trafficking to the MVB could have a role for Comm localisation. The PPCY motif in the Comm sorting domain is similar to motifs recognised by the ESCRT-II complex suggesting this may provide a link to other components of the ESCRT machinery. Comm is also found associated with Shrub/CHMP4/Scf7, a component of ESCRT-III found at sorting endosomes. However ubiquitination of Comm is not necessary for its activity in commissural neurons and the ESCRT proteins are best characterised for their ability to direct the trafficking of ubiquitinated proteins. Although it is been suggested that ESCRT proteins may also traffic non-ubiquitinated cargo in Drosophila, where roles for vps25 and vps23 have been identified for trafficking of Notch [51]–[53]. Comm localisation may utilise a number of redundant mechanisms and this can be tested in the future. It has emerged that axon navigation at the midline does involve several redundant mechanisms [1], [54]and that Robo can be regulated through both Comm dependent and independent mechanisms [55] suggesting a high level of redundancy in the processes underlying axon guidance.
